# Supplementary material for: High Nucleotide Substitution Rates Associated with Retrotransposon Proliferation Drive Dynamic Secretome Evolution in Smut Pathogens
Source: Microbiol Spectr. 2022 Aug 16;10(5):e00349-22. doi: 10.1128/spectrum.00349-22 (PMC9603552; doi:10.1128/spectrum.00349-22)
Supplement: Supplemental file 1 — Supplemental material. Download spectrum.00349-22-s0001.pdf, PDF file, 2.2 MB [file spectrum.00349-22-s0001.pdf]

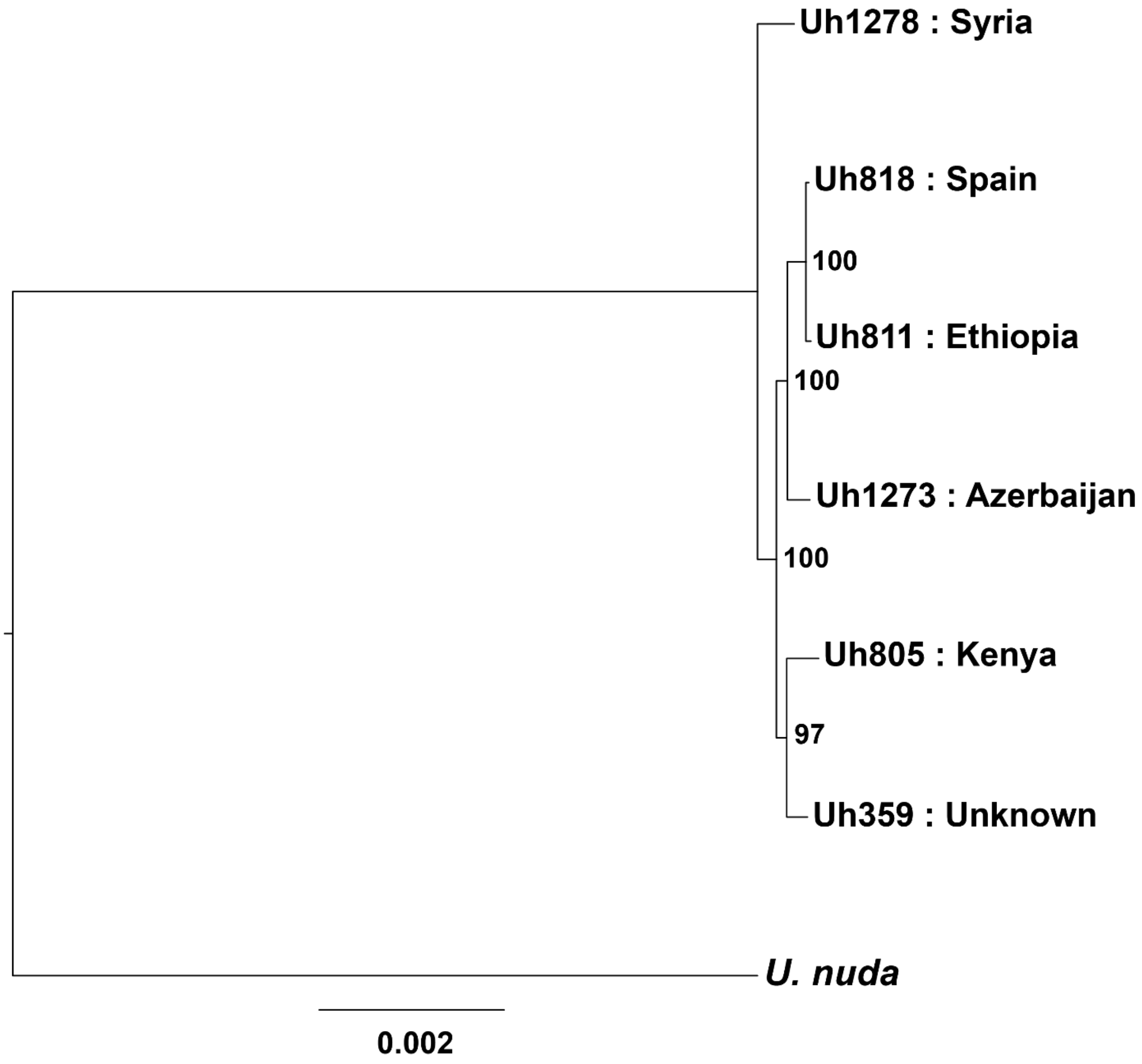

Figure S1 Phylogenetic relationship between *Ustilago hordei* lineages based on Benchmarking Universal Single-Copy Orthologs (BUSCOs). In total, 1,692 BUSCOs were used for tree construction. Homologous BUSCO protein sequences were aligned using MAFFT and then concatenated for tree construction using RAxML with substitution model "PROTGAMMAWAG". *U. nuda* was used as on outgroup species to root the tree. The robustness of the trees was assessed using 100 bootstrap replicates.

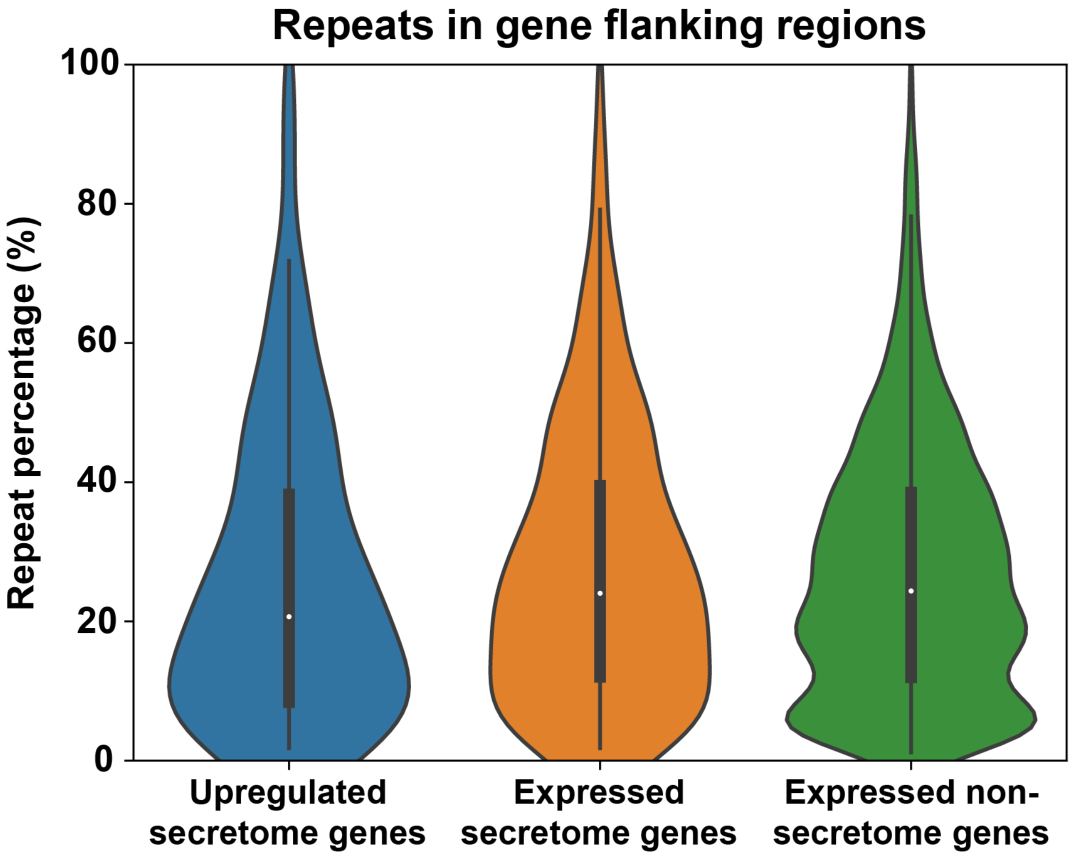

Figure S2 Comparison of repeat content of gene flanking regions between expressed secretome genes and other expressed genes. Upregulated means a significantly higher expression *in planta* compared to growth in axenic culture. In total, 20 kb sequences on each side of the genes (40 kb in total) were considered as flanking regions. Significant differences were calculated with a two-sided T-test. No significant differences with *p*-value < 0.01 were found.

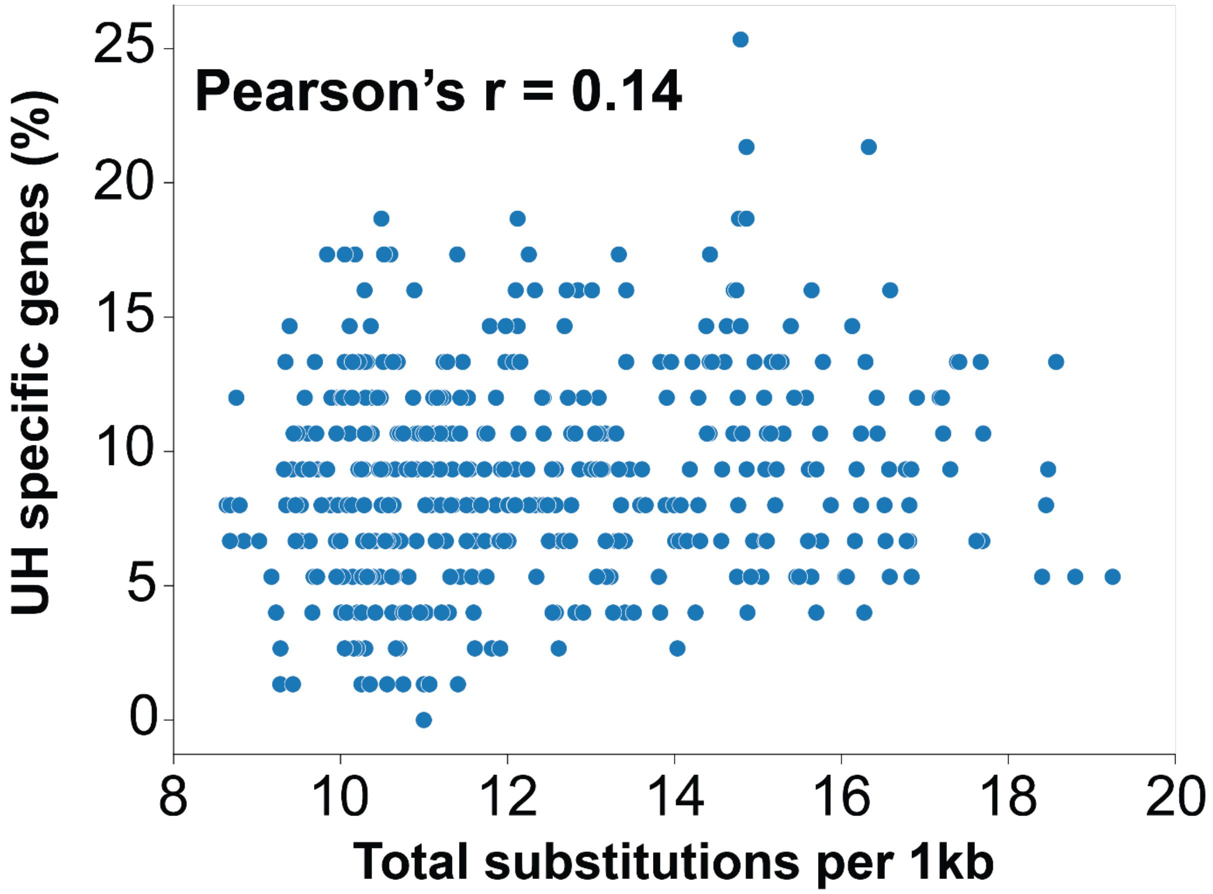

Figure S3: Correlation between median nucleotide substitution level and fraction *U. hordei* (UH) specific genes for ortholog windows. Ortholog windows of 75 UH genes with a sliding step of 10 were used to determine the number of substitutions with *U. nuda*. UH specific genes do not have an ortholog in *U. maydis*.

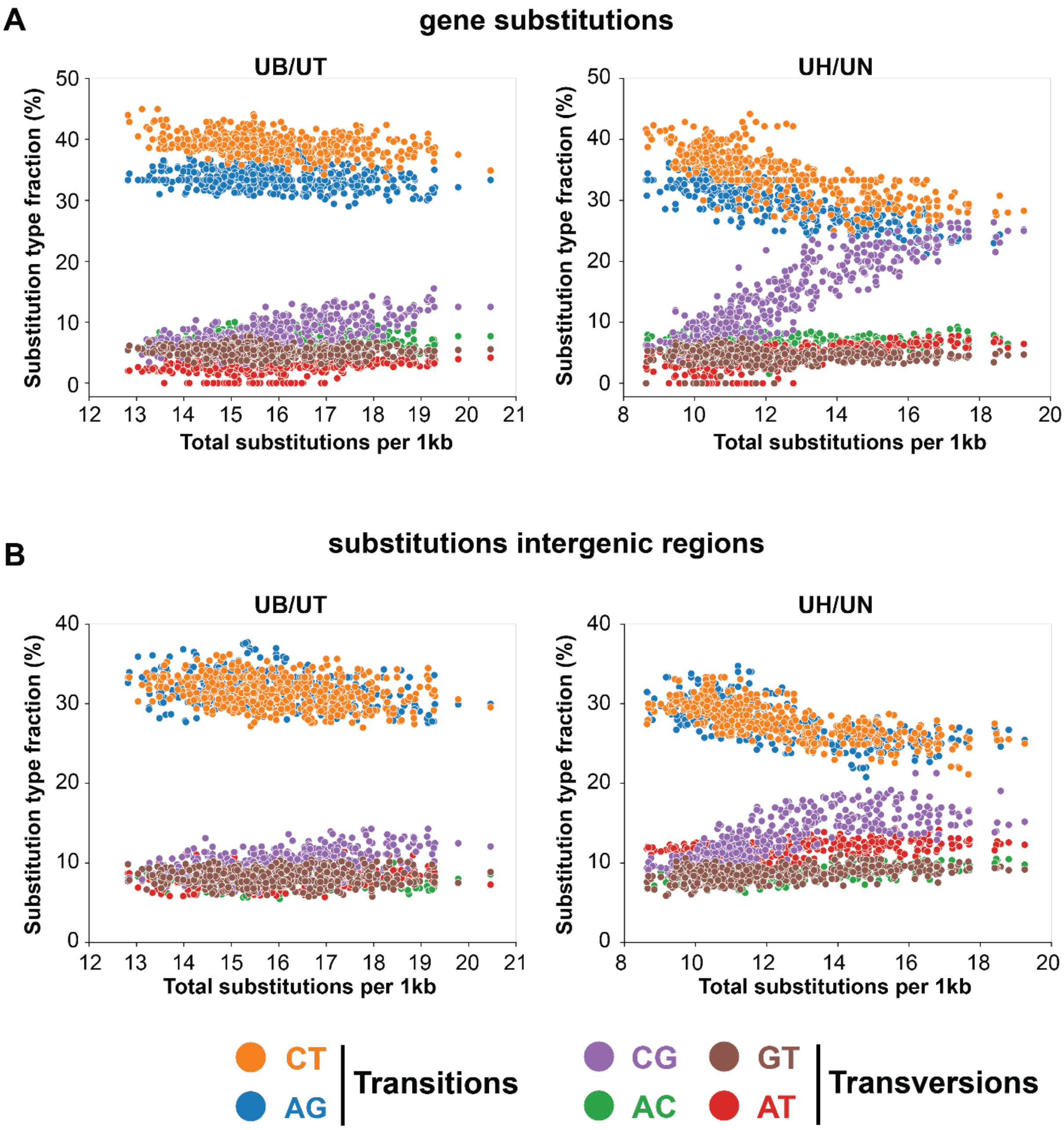

Figure S4: Comparison of nucleotide substitution type fractions for *U. brachipodii-distachyi*/*U. tritici* (UB/UT) and *U. hordei*/*U. nuda* (UH/UN) ortholog windows. The nucleotide substitutions were calculated for windows of 75 genes with a sliding step of 10. The x-axis consistently displays the total substitutions per 1 kb for these windows. (**A**) The y-axis depicts the fraction of every substation type (CT, AG, CG, AC, GT, AT) of ortholog windows. (**B**) The y-axis depicts the fraction of every substation type for the intergenetic regions of ortholog windows.

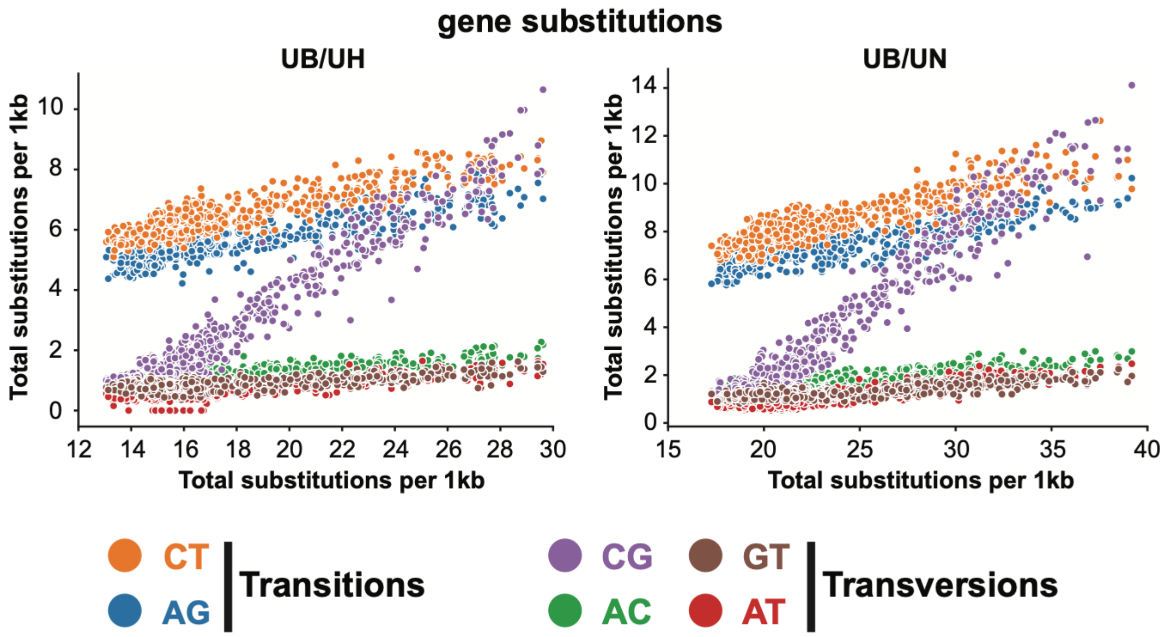

Figure S5: Comparison of nucleotide substitution regimes for *U. brachipodii-distachyi*/*U. hordei* (UB/UH) and *U. brachipodii-distachyi*/*U. nuda* (UB/UN) ortholog windows. The nucleotide substitutions were calculated for windows of 75 genes with a sliding step of 10. The x-axis consistently displays the total substitutions per 1 kb for these windows. The y-axis depicts the median number of every substation type (CT, AG, CG, AC, GT, AT) of ortholog windows.

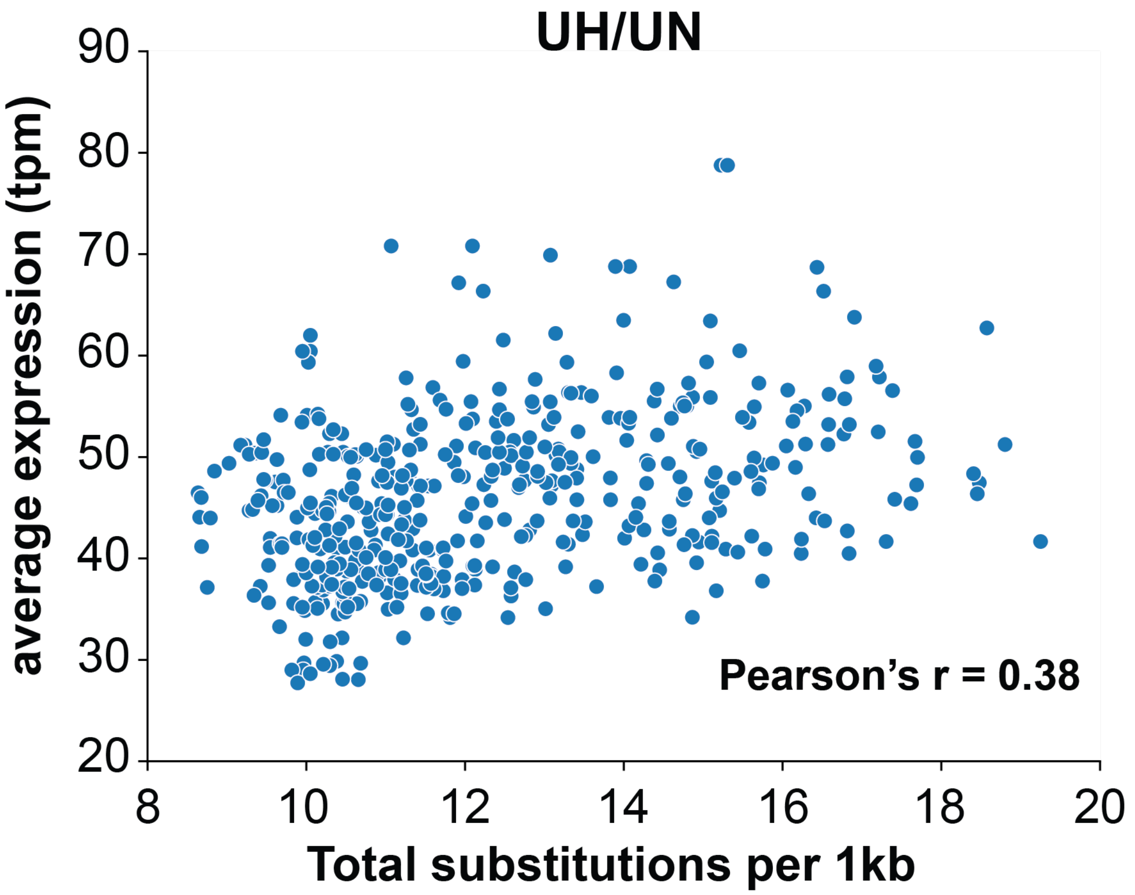

Figure S6: High local nucleotide substitution levels apply on genome regions with transcriptionally active genes. The relation between the median number of nucleotide substitutions and the median expression level for ortholog windows of 75 genes with a sliding step of 10. Substitution levels were determined for *U. horei* genes in comparison to *U. nuda* orthologs. Expression levels for every *U. hordei* gene were calculated as the average transcripts per million (tpm) for the six RNA seq samples i.e. three samples each of in liquid culture medium and *in planta* grown *U. hordei*.

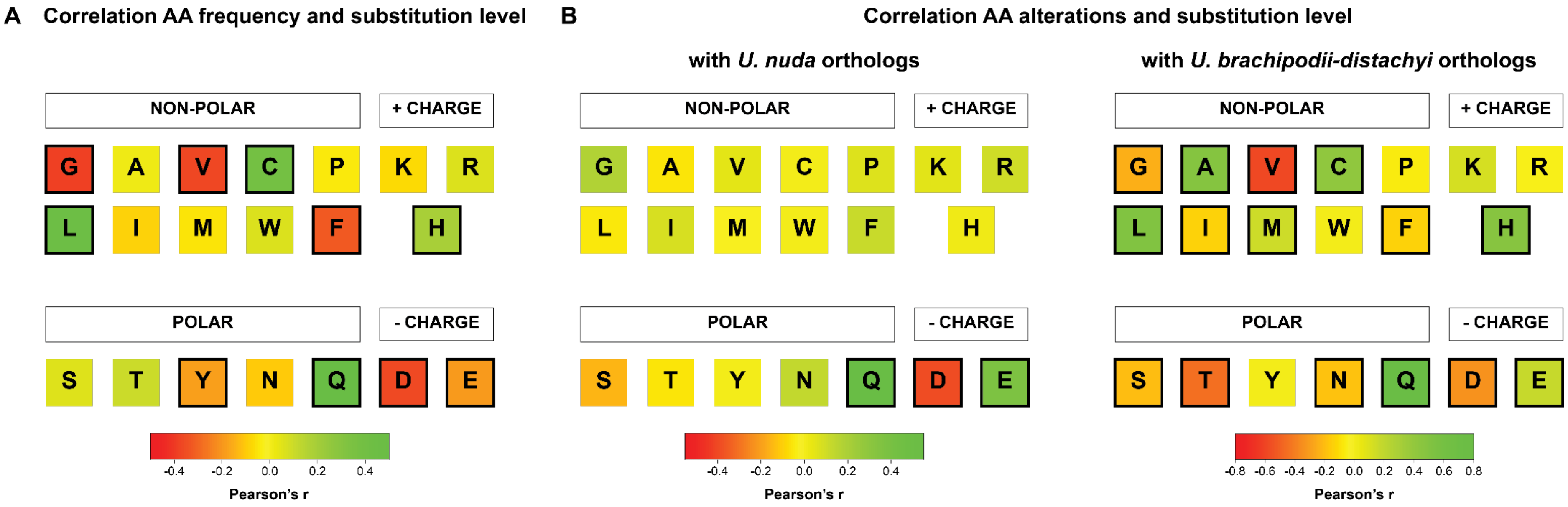

Figure S7: Correlations between the nucleotide substitution levels and the encoded amino acid composition of genes. Correlations were calculated for windows of 75 *U. hordei* genes with a sliding step of 10. Significant correlations, with *p*-value < 0.01, are indicated by a black edge around the square. (**A**) Correlations between amino acid compositions of encoded *U. hordei* proteins and the number of nucleotide substitutions with *U. nuda*. (**B**) Correlations between amino acid alternations for encoded *U. hordei* proteins and the number of nucleotide substitutions using *U. nuda* and *U. brachipodii-distachyi* orthologs as comparison.

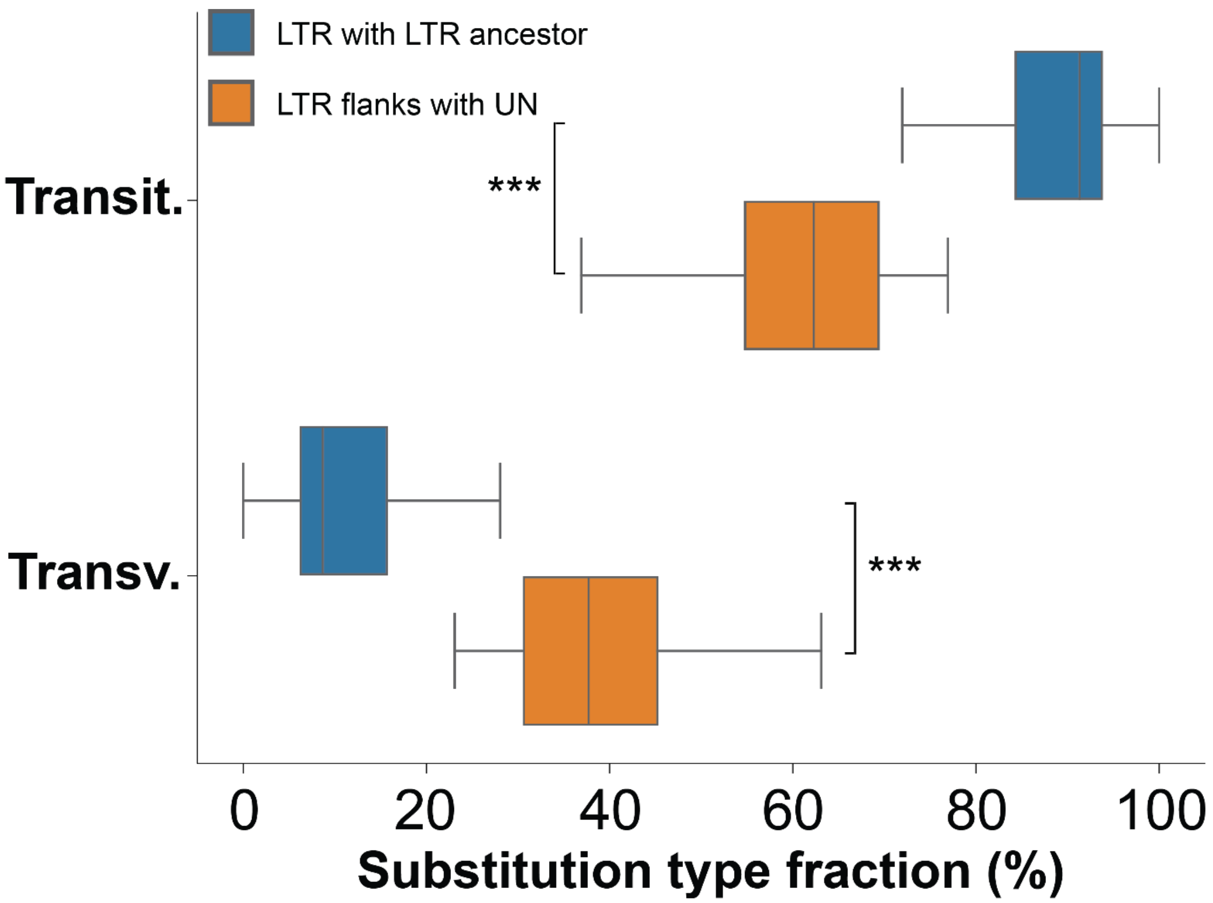

Figure S8: Fractions of transitions and transversion of recently proliferated *U. hordei* long terminal repeat retrotransposons (LTR-RTs) and their flanking regions. The fraction of transitions and transversions between recently proliferated *U. hordei* LTR-RTs and their ancestors were determined. Fractions of the 20 kb flanking regions (40 kb in total) of the LTR-RT with *U. nuda* (UN) were also determined. Significant differences between LTR and flanking regions were determined for transitions and transversions separately with an unequal variance t-test. ***: p-value < 0.001.

Table S1 Characteristics of the mating-type loci of smut species with a bipolar mating-type system.

Species	Strain	Mating-type	Length* (kb)	Repeat content Genome wide (%)	Repeat content *MAT* locus (%)	LTR-RT§ Genome wide (%)	LTR-RT§ *MAT* locus (%)
U. hordei	Uh359	MAT1	564	38.2	78.2	19.5	33.1
	Uh805	MAT1	560	35.3	77.1	16.8	32.5
	Uh811	MAT1	536	36.4	76.1	17.6	31.6
	Uh818	MAT1	536	36.5	76.2	17.3	29.8
	Uh1273	MAT1	545	38.9	77.5	19.1	32.8
	Uh1278	MAT2	472	36.0	75.4	17.3	29.6
U. nuda	DE_29490	MAT1	285	22.6	66.5	7.2	33.5
U. brachipodii-distachyi	UB2112	MAT1	190	17.0	49.1	2.3	5.4
U. tritici	Ut_3	MAT1	249	16.4	38.6	2.3	2.3

*The length that is spanned by *bEast*, *bWest*, the pheromone receptor (*pra*) and the pheromone (*mfa*) genes.

§ Only long terminal repeat retrotransposons (LTR-RTs) larger than 500 bp were annotated.

Table S2. Transposable elements annotation in various smut genome assemblies.

Species	*U. hordei*						*U. nuda*	*U. brachipodii-distachyi*	*U. tritici*	*U. loliicola*	*U. maydis*	*S. reilianum*
Strain	Uh359	Uh805	Uh811	Uh818	Uh1273	Uh1278	DE_29490	UB2112	Ut_3	Us_530	521	SRS1_H2-8
Class I TEs (kb)[§]	5,625	4,611	4,985	4,897	5,663	4,940	1,786	672	739	102	199	5
LTR (kb)	5,272	4,326	4,615	4,549	5,208	4,607	1,537	463	462	9	185	5
LARD (kb)	126	54	213	136	233	104	106	39	43	65	11	0
TRIM (kb)	4	16	11	11	11	12	4	1	4	0	0	0
DIRS (kb)	163	117	59	117	97	122	98	135	119	0	0	0
LINE (kb)	8	0	0	5	22	7	9	4	71	27	3	0
SINE (kb)	0	1	0	0	1	2	1	0	1	1	0	0
Class II TEs (kb)[§]	781	746	708	791	731	692	395	473	285	482	5	103
TIR (kb)	604	619	589	667	612	622	342	418	242	322	5	100
MITE (kb)	12	13	11	18	13	12	11	9	5	88	0	2
Crypton (kb)	31	30	34	34	23	30	42	45	38	0	0	0
Maverick (kb)	38	11	22	21	31	15	0	0	0	0	0	0
Helitron (kb)	92	73	47	45	51	3	0	0	0	72	0	0

[§] Only repetitive sequences that were larger than 500 bp were classified.
